# Supplementary material for: The causal role of circulating inflammatory markers in osteoporosis: a bidirectional Mendelian randomized study
Source: Front Immunol. 2024 Jul 18;15:1412298. doi: 10.3389/fimmu.2024.1412298 (PMC11291241; doi:10.3389/fimmu.2024.1412298)
Supplement: Supplementary file 1 [file DataSheet_1.zip › supplementary material/CIM-OSTEOPOROSIS /GCST90274799.csv_leave_one_out.pdf]

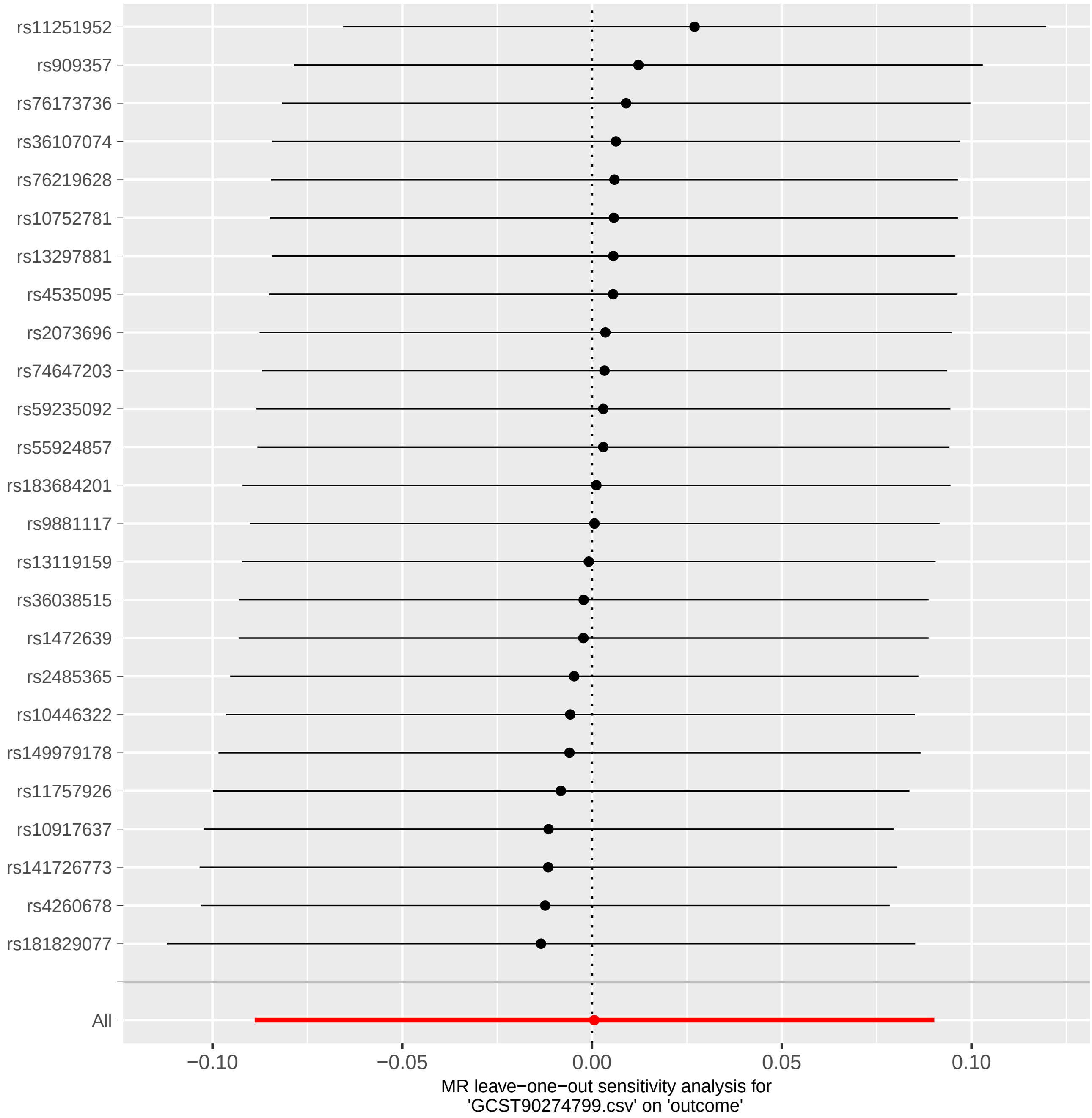
rs11251952
rs909357
rs76173736
rs36107074
rs76219628
rs10752781
rs13297881
rs4535095
rs2073696
rs74647203
rs59235092
rs55924857
rs183684201
rs9881117
rs13119159
rs36038515
rs1472639
rs2485365
rs10446322
rs149979178
rs11757926
rs10917637
rs141726773
rs4260678
rs181829077
All
−0.10
−0.05
0.00
0.05
0.10
MR leave−one−out sensitivity analysis for
'GCST90274799.csv' on 'outcome'
